# Antimicrobial activity of topcoat formulation based on synthesized new cyclodiphosph(V)azane derivatives as a biocide for protective coatings

**DOI:** 10.1038/s41598-026-52099-1

**Published:** 2026-05-19

**Authors:** H. Abd El-Wahab, Narmeen G. El khashab, Salwa A. H. Albohy, Moustafa M. G. Fouda, Mohamed H. Sharaf, Mahmoud M. Fayad, Carmen M. Sharaby

**Affiliations:** 1 Chemistry Department, Faculty of Science (Boys), Al-Azhar University, Nasr City, Cairo Egypt; 2 Chemistry Department, Faculty of Science (Girls), Al-Azhar University, Nasr City, Cairo Egypt; 3 Pre-Treatment and Finishing of Cellulose Based Textiles, Textile Research and Technology Institute (TRT), National Research Center, 33- El-Buhouth St, Dokki, Cairo, Egypt; 4 Microbiology Department, Faculty of Science (Boys), Al-Azhar University, Nasr City, Cairo Egypt; 5 Production department, Drilling fluid lab Nasr City, Egyptian Petroleum Research Institute, Nasr City, 11727 Cairo Egypt

**Keywords:** Cyclodiphosph(V)azane, Metal complexes, Antimicrobial coatings, Paint additives, Physico-mechanical properties, Antibacterial activity, Surface protection, Biotechnology, Chemistry, Microbiology

## Abstract

Novel cyclodiphosph(V)azane sulfonamide ligands and their corresponding Cu²⁺ and Cd²⁺ metal complexes were synthesized and evaluated as antimicrobial additives for coating applications. The structures of the prepared compounds were confirmed using standard spectroscopic techniques. The synthesized compounds were incorporated into paint formulations, and the resulting coatings were systematically assessed for their mechanical, physical, and antimicrobial properties. The modified coatings exhibited enhanced physico-mechanical performance, with gloss values of 80–95, hardness ranging from 7 H to 9 H, adhesion improved from 4B to 5B, and impact resistance increased from 1.3 to 2.5 J. Antimicrobial activity was evaluated using the agar well diffusion method against representative Gram-positive and Gram-negative bacteria, as well as fungal strains. The cadmium complex demonstrated the highest activity, with inhibition zones ranging from 31.8 to 46.0 mm, while the copper complex showed moderate activity and the free ligand exhibited selective effects. Although incorporation into the coating matrix led to a reduction in antimicrobial activity, the coatings retained appreciable effectiveness against selected strains. The results indicate that cyclodiphosph(V)azane-based metal complexes are promising multifunctional additives for antimicrobial coatings, combining improved mechanical performance with significant biological activity.

## Introduction

The increasing incidence of microbial contamination and its associated health and economic impacts have intensified the demand for effective antimicrobial materials. Surface coatings with antimicrobial functionality represent a practical and widely applicable solution for limiting microbial growth and biofilm formation in both industrial and healthcare environments^1,2^. Antimicrobial coatings are typically formulated by incorporating active agents such as organic biocides, metal-based compounds, or nanomaterials into polymer matrices^3–7^. These agents provide prolonged protection by inhibiting the growth of bacteria, fungi, and algae on coated surfaces. In coating technology, biocides are generally classified into in-can preservatives, which protect the formulation during storage, and film preservatives, which act on the applied coating to prevent microbial colonization^8–10^. Additives are essential components in coating formulations, often designed to impart specific functionalities, including mechanical reinforcement, durability, and resistance to environmental degradation. In recent years, significant attention has been directed toward the development of multifunctional additives capable of simultaneously enhancing mechanical properties and providing antimicrobial activity^11–14^. Cyclodiphosph(V)azane derivatives have emerged as promising candidates in this context due to their structural versatility and potential biological activity. Previous studies have demonstrated their application in epoxy and polyurethane coatings^15^, where they exhibit moderate antimicrobial performance along with additional functionalities such as flame retardancy. Furthermore, metal complexes derived from cyclodiphosph(V)azane ligands have enhanced biological properties, including antibacterial and anticancer activities^16–19^, highlighting the importance of metal coordination in improving efficacy^20–23^. In addition to cyclodiphosph(V)azane systems, other classes of compounds—such as coumarins, arylhydrazones, benzodiazepines, and benzothiophenes—have been successfully explored as antimicrobial coating additives. These materials have demonstrated the ability to improve both the biological and mechanical performance of coatings, reinforcing the importance of designing multifunctional systems^24–27^. In the present study, new cyclodiphosph(V)azane sulfonamide ligands and their metal complexes were synthesized and characterized. These compounds were incorporated into coating formulations to evaluate their influence on mechanical and physical properties, as well as their antimicrobial performance. The objective is to develop efficient antimicrobial coatings with enhanced durability and functionality for advanced applications.

## Experimental part

### Materials

Copper chloride dihydrate (CuCl₂·2 H₂O) and cadmium chloride dihydrate (CdCl₂·2 H₂O) were used as received. *p*-Chloroaniline and phosphorus pentachloride (PCl₅) were also employed in this study. All chemicals were of analytical grade and used without further purification. Solvents were obtained from local and international suppliers, including BDH and Merck. A commercial top coating was supplied by Pachin Company, Egypt.

### Synthesis of hexachlorocyclodiphosph(V)azane ligand (L), and its metals complexes

**Scheme 1:**
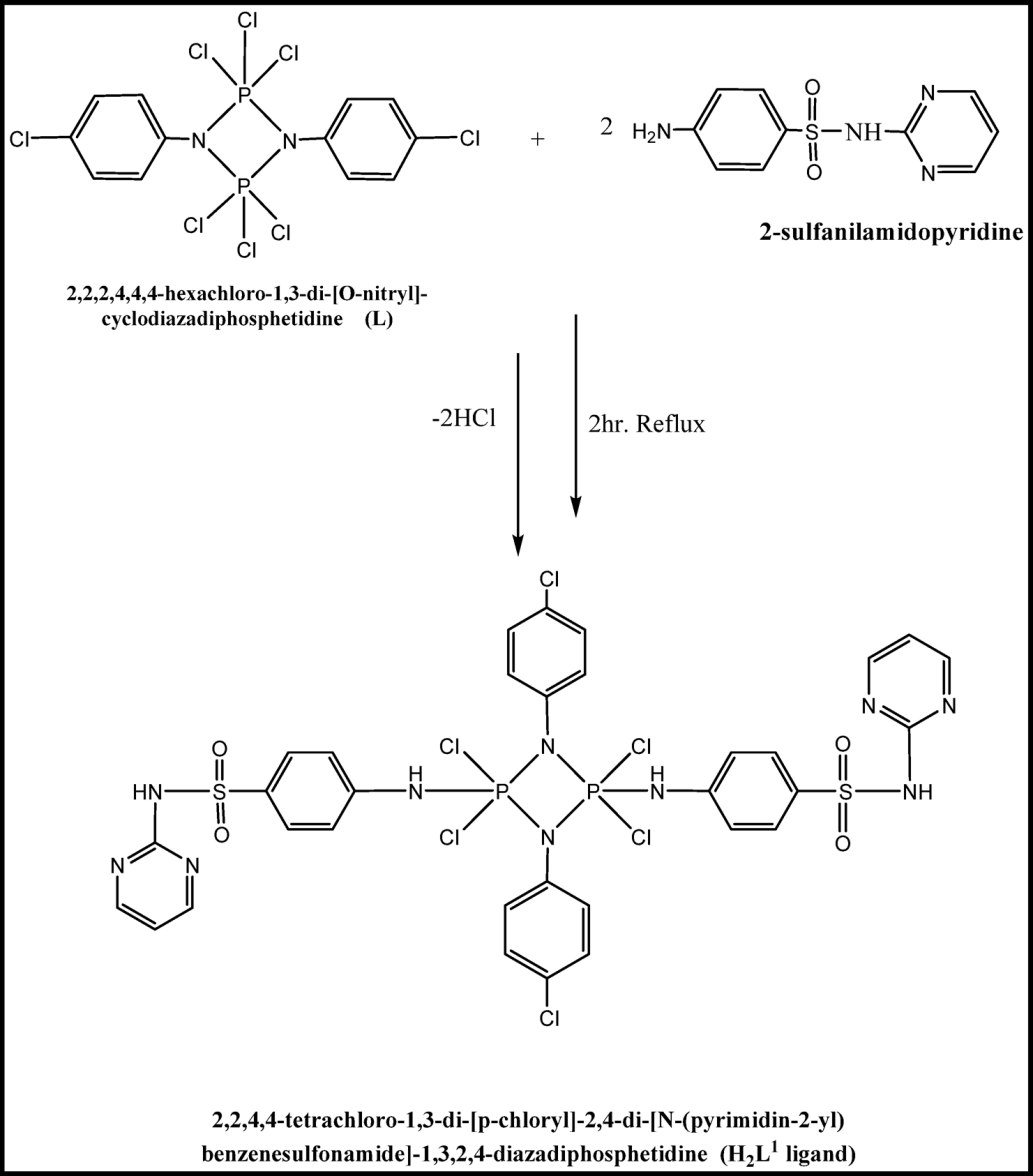
Synthesis of substituted cyclodiphospha(V)zane of free ligand (L).

**Scheme 2:**
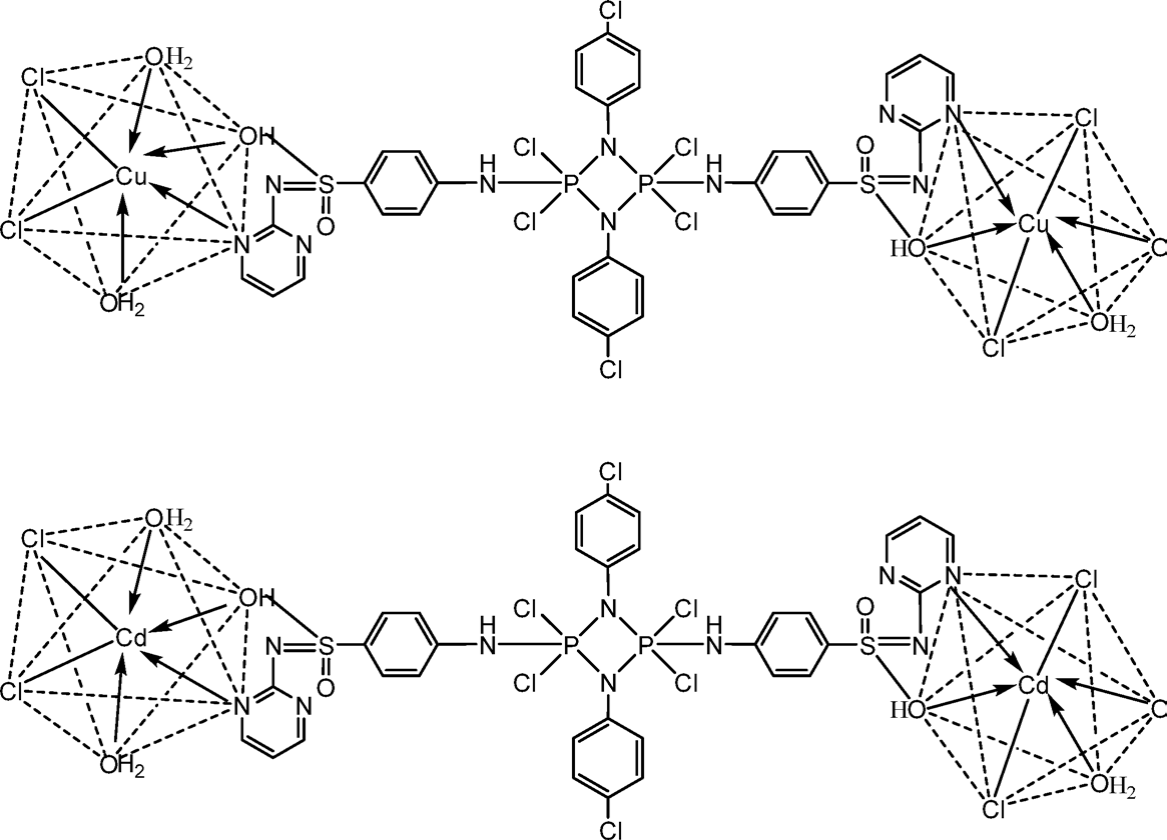
Suggested structure of the newly obtained free ligand (L), and its Cu²⁺ and Cd²⁺ metal complexes.

The hexachlorocyclodiphosph(V)azane ligand (L) and its Cu²⁺ and Cd²⁺ metal complexes were synthesized according to our previous report. In the present study, ligand synthesis was modified by substituting *o*-nitroaniline with *p*-chloroaniline. As shown in Scheme 1, and 2.

The resulting ligand (L) was obtained as a brownish-yellow solid with a melting point of 200 °C and a yield of 70% (4 g).

#### Instrumentation

Fourier-transform infrared (FT-IR) spectra was recorded using a PerkinElmer Model 297 IR spectrometer employing the KBr pellet technique. Elemental microanalysis for carbon (C), hydrogen (H), nitrogen (N), and sulfur (S) was carried out using a PerkinElmer 2408 CHN analyzer.

Proton nuclear magnetic resonance (^1^H NMR) spectra were obtained in DMSO-d₆ at room temperature on a Varian FT-300 MHz spectrometer, using tetramethylsilane (TMS) as an internal standard.

Ultraviolet–visible (UV–Vis) spectra were measured with a PerkinElmer Lambda 3B UV–Vis spectrophotometer. Mass spectra were recorded using a direct insertion probe (DIP) on a Shimadzu GC–MS QP 100 EX mass spectrometer (Japan) over a temperature range of 50–1000 °C.

The structural and morphological features were examined using a JEOL JSM-T330A scanning electron microscope (SEM) operated at an accelerating voltage of 30 kV.

### Antimicrobial activity assessment

#### Test microorganisms

The antimicrobial activity was evaluated against a panel of microorganisms, including *Staphylococcus aureus* (MRSA), *Bacillus cereus*, *Escherichia coli*, *Acinetobacter baumannii*, *Candida albicans*, and *Aspergillus niger*. All strains were obtained from the bacteriology laboratory, Department of Botany and Microbiology, Faculty of Science, Al-Azhar University.

Bacterial isolates were maintained on Mueller–Hinton agar (MHA), while *C. albicans* and *A. niger* were maintained on Sabouraud dextrose agar (SDA). Prior to testing, all isolates were subcultured to ensure viability and purity^28^.

#### Preparation of inoculum

Fresh bacterial colonies were suspended in sterile saline solution (0.85% NaCl) and adjusted to match the 0.5 McFarland standard (approximately 1–1.5 × 10⁸ CFU/mL). The inoculum of *C. albicans* was prepared to a final concentration of approximately 1 × 10⁶ CFU/mL. *A. niger* conidial suspensions were obtained from 5–7-day-old cultures grown on SDA. All microbial suspensions were used within 15 min of preparation to maintain consistency and viability^28^.

#### Agar well diffusion assay

The antimicrobial activity of copper and cadmium complexes, as well as their corresponding ligand—either alone or incorporated into paint—was evaluated using the agar well diffusion method.

Sterile MHA or SDA plates were uniformly inoculated using a sterile cotton swab to produce a confluent lawn of microbial growth. Wells of 6 mm diameter were aseptically prepared using a sterile cork borer. Subsequently, 100 µL of each test sample was carefully introduced into each well.

The plates were allowed to stand at room temperature for 30 min to permit diffusion of the test compounds into the agar medium^28^.

Bacterial cultures were incubated at 37 °C for 18–24 h, while fungal cultures (*C. albicans* and *A. niger*) were incubated at 28 °C for 48–72 h.

Following incubation, the diameter of the inhibition zones (including the well diameter) was measured in millimeters using a digital caliper. All experiments were conducted in triplicate (or at least duplicate), and results were expressed as mean ± standard error of the mean (SE).

Sharaf et al.,2026^29^ described a procedure to assess the MIC values of the different compounds against the tested bacteria. To prepare 1000, 500, 500, 250, 125, 62.5, 31.25, and 15.6 µg/ml, 100 µl of each concentration of compound was added to sterile microtiter plate wells that contained 100 µl of double-strength Mueller Hinton broth (MHB). All wells except the sterility test (ST), which contained sterile DH2O and MHB to guarantee sterility, were filled with a 20 µl bacterial cell suspension. To verify that the broth could sustain Microbial growth, MHB and bacterial suspension were added to the positive control wells, which were then incubated for one day at 37 °C for bacteria and incubated for two day at 28 °C for fungi. After incubation, each well received 30 µl of a 0.02% (wt/v) resazurin solution (HiMedia), and the plates were then incubated for an additional 4 h. The growth control wells’ color changes to either pink, red, or purple confirmed adequate growth of the isolate, while no color change in the sterile control wells indicated the absence of contamination^30^.

## Results and discussion

### Physical properties and elemental analysis

The physical properties and molar conductivity of the ligand (L) and its corresponding metal complexes are summarized in Table 1.

Table 1 and Scheme 2 are consistent with the observed results. The complexes are soluble in dimethyl sulfoxide (DMSO) and dimethylformamide (DMF), but insoluble in water. Molar conductance (ΛM) measurements of the complexes were carried out at a concentration of.

10⁻³ M using DMF as the solvent. The obtained values indicate that the Cu²⁺ and Cd²⁺ metal complexes exhibit non-electrolytic behavior. As shown in Table 1, the conductivity values range from 0.61 to 4.14 Ω⁻¹ cm² mol⁻¹, supporting their non-electrolytic nature and the neutrality of the coordination sphere. These findings suggest that the anions are coordinated within the inner sphere of the metal complexes.

As shown in Table 2. The Elemental analysis (%) was performed to verify the proposed chemical structures of the ligand and its metal complexes. The analytical data presented in Table 2 shows good agreement between the experimental and calculated values, supporting the proposed compositions.

**Fig. 1:**
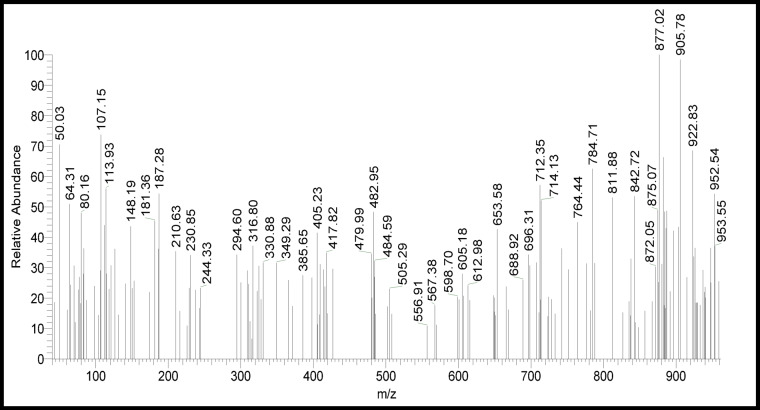
Mass fragmentation of free ligand (L).

Also, as shown in Fig. 1, the molecular formula, C₃₂H₂₆Cl₆N₁₀O_4_P₂S₂, was further supported by mass spectrometry, which exhibited a base peak at m/z = 877.02 (100%) and a molecular ion peak at m/z = 953 (37.03%). This agrees well with the suggested formula which is in Scheme 1.

**Table 1:** Physical properties and molar conductivity of ligand (L) and its metal complexes.

Compound	Formula (M.Wt, g mol⁻¹)	M.*P*. (°C)	Colour	Yield (%)	Λm (Ω⁻¹ cm² mol^− 1^)
Ligand (L)	C₃₂H₂₆Cl₆N₁₀O₄P₂S₂ (953)	200	Pale yellow	70	3.2
Cu²⁺ complex	C₃₂H₃₄Cl₁₀Cu₂N₁₀O₈P₂S₂ (1294)	> 300	Dark green	45	4.14
Cd²⁺ complex	C₃₂H₃₄Cd₂Cl₁₀N₁₀O₈P₂S₂ (1392)	> 300	Faint yellow	32	0.61

**Table 2:** Elemental analysis (%) of ligand (L) and its metal complexes.

Compound	C	H	*N*	Cl	S	*P*	Metal
Ligand (L)	40.31 (40.35)	2.23 (2.74)	13.42 (14.69)	22.35 (22.35)	6.30 (6.72)	6.49 (6.49)	–
Cu²⁺ complex	29.71 (29.69)	2.33 (2.65)	10.66 (10.82)	25.61 (27.39)	4.90 (4.95)	4.95 (4.79)	9.82 (9.82)
Cd²⁺ complex	25.89 (27.61)	2.11 (2.46)	10.05 (10.06)	25.46 (25.47)	4.61 (4.61)	4.45 (4.45)	16.15 (16.15)

Values in parentheses are calculated. Λm = molar conductivity measured in DMF at room temperature.

### Magnetic characteristics and the electronic spectrum

**Fig. 2:**
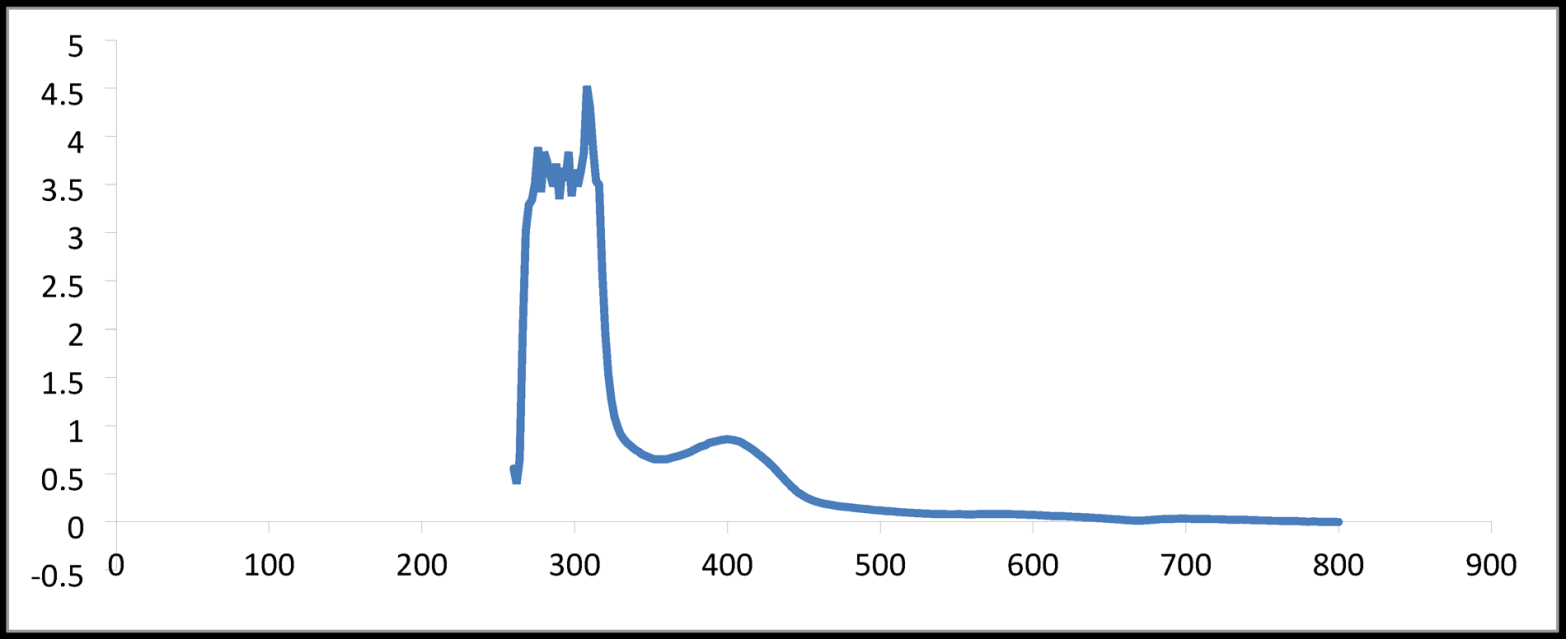
UV-vis spectrum.

Based on tabulated results as shown in Table 3 and presented in Fig. 2. The electronic spectrum of the ligand (L) and its Cu²⁺ and Cd²⁺ metal complexes were analyzed to determine their electronic transitions and infer structural features. The free ligand exhibited characteristic band at (270 nm) for phosphazo four membered ring with dimeric structures π→π* (330 nm) and n→π* (388 nm) transitions associated with the phosphazo and aromatic systems. Upon complexation, these bands underwent slight blue shifts, indicating coordination of nitrogen donor atoms to the metal centers. The Cu²⁺ complex displayed an additional band at 418 nm attributed to ligand-to-metal charge transfer (L→MCT), confirming electron donation from the ligand to the metal. A d–d transition at 528 nm further supported the octahedral geometry of the Cu²⁺center, consistent with the measured magnetic moment of 1.4 B.M., indicative of one unpaired electron. In contrast, the Cd²⁺ complex, with a d¹⁰ electronic configuration, showed no d–d transitions, and its spectrum was dominated by ligand-based transitions, also suggesting an octahedral coordination environment. Overall, the spectral data corroborates the successful coordination of the ligand to both Cu²⁺ and Cd²⁺, with distinct electronic signatures reflecting the metal’s electronic configuration^16,20,21,31–36^.

**Table 3:** Electronic spectral data of the ligand and its Cu(II) and Cd(II) complexes.

Compound	Phosphazo Ring (nm)	π→π* (nm)	*n*→π* (nm)	L→MCT (nm)	d–d Transition (nm)	µeff (B.M.)	Geometry
ligand (L)	270	330	388	–	–	–	–
Cu²⁺ complex	276	310	324	418	528	1.4	Octahedral
Cd²⁺ complex	274	314	378	d¹⁰ (no d–d)	–	–	Octahedral

### IR characterization

**Fig. 3:**
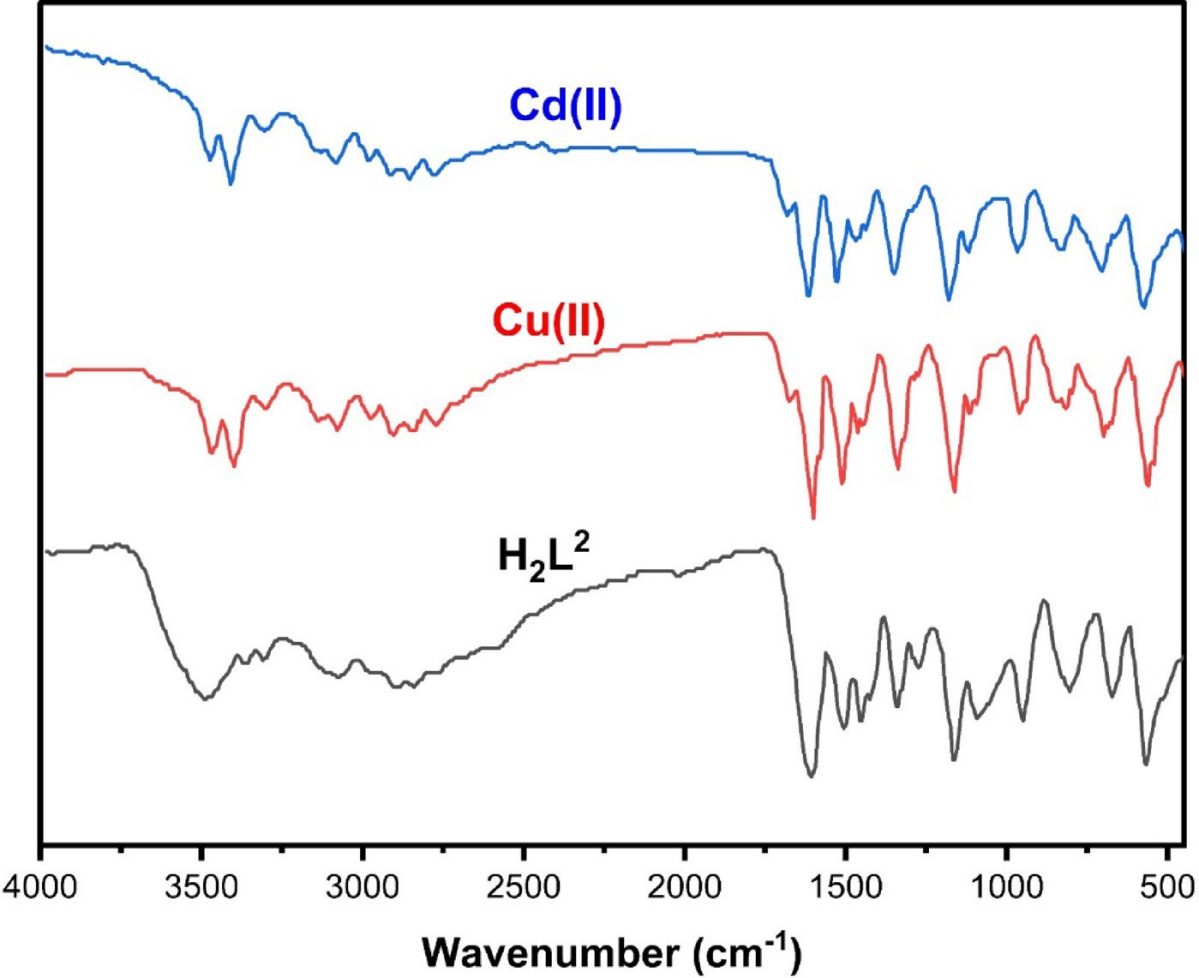
IR spectra of -free ligand and its Cu²⁺, Cd²⁺ metal complexes.

As shown in Table 4; Fig. 3, we can notice that the IR spectrum of the free ligand exhibits characteristic bands at 1154 cm⁻¹ and 662 cm⁻¹, corresponding to υ(P–N) and υ(P–Cl) vibrations, respectively^20,21^. Additional bands at 3421 cm⁻¹ and 1618 cm⁻¹ are assigned to υ(NH) and υ(C = N), confirming the presence of amino and azomethine functionalities. Upon complexation with Cu²⁺ and Cd²⁺, significant changes in the IR spectra confirm metal coordination. The ν(C = N) stretching frequency shifts from 1618 cm⁻¹ in the ligand to 1585–1586 cm⁻¹ in the complexes, while the asymmetric ν(SO₂) band moves from 1344 cm⁻¹ to 1322–1324 cm⁻¹, indicating coordination through the azomethine nitrogen and sulfonyl oxygen. The ν(NH/OH) band shows slight broadening, reflecting hydrogen bonding and possible weak coordination, whereas the ν(P–N) and ν(P–Cl) bands remain largely unchanged, suggesting that these groups are not involved in metal binding. The presence of coordinated water is confirmed by bands at 842 cm⁻¹ Cu²⁺ and 799 cm⁻¹ Cd²⁺, and metal-ligand bonding is further evidenced by ν(M–O) (547–550 cm⁻¹) and ν(M–N) (412 cm⁻¹) vibrations. Collectively, these spectral observations demonstrate that free ligand (L) coordinates to Cu²⁺ and Cd²⁺ through the azomethine nitrogen and oxygen atoms, with water molecules completing the coordination sphere, while the phosphorous-linked groups remain uncoordinated.

**Table 4:** IR spectral data (cm⁻¹).

Compound	ν(NH/OH)	ν(SO₂) asym	ν(SO₂) sym	ν(C = *N*)	ν(*P*–*N*)	ν(*P*Cl)	ν(H₂O) coord	ν(M–O)	ν(M–*N*)
ligand (L)	3421 sh	1344 sh	1087 sh	1618 sh	1154 sh	662 sh	–	–	–
Cu²⁺ complex	3423 br	1322 m	1092 br	1585 sh	1154 sh	684 m	842 m	547 m	412 m
Cd²⁺ complex	3424 sh	1324 sh	1091 sh	1586 sh	1155 sh	675 m	799 w	550 m	412 m

sh = sharp, m = medium, br = broad, w = weak.

### ^1^H NMR data of free ligand and Cd(II) metal complex

**Fig. 4:**
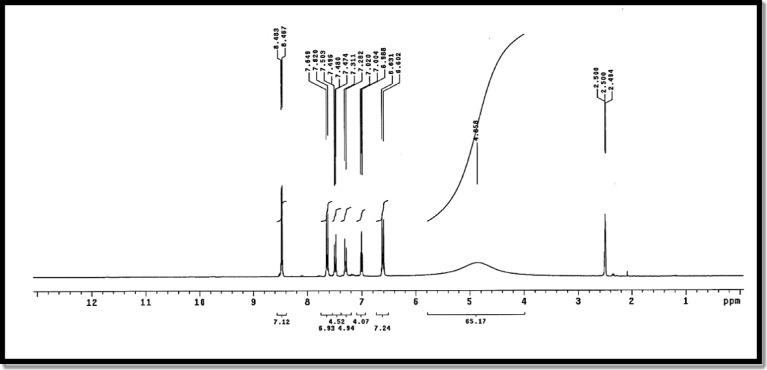
^1^H NMR spectrum of free ligand.

**Fig. 5:**
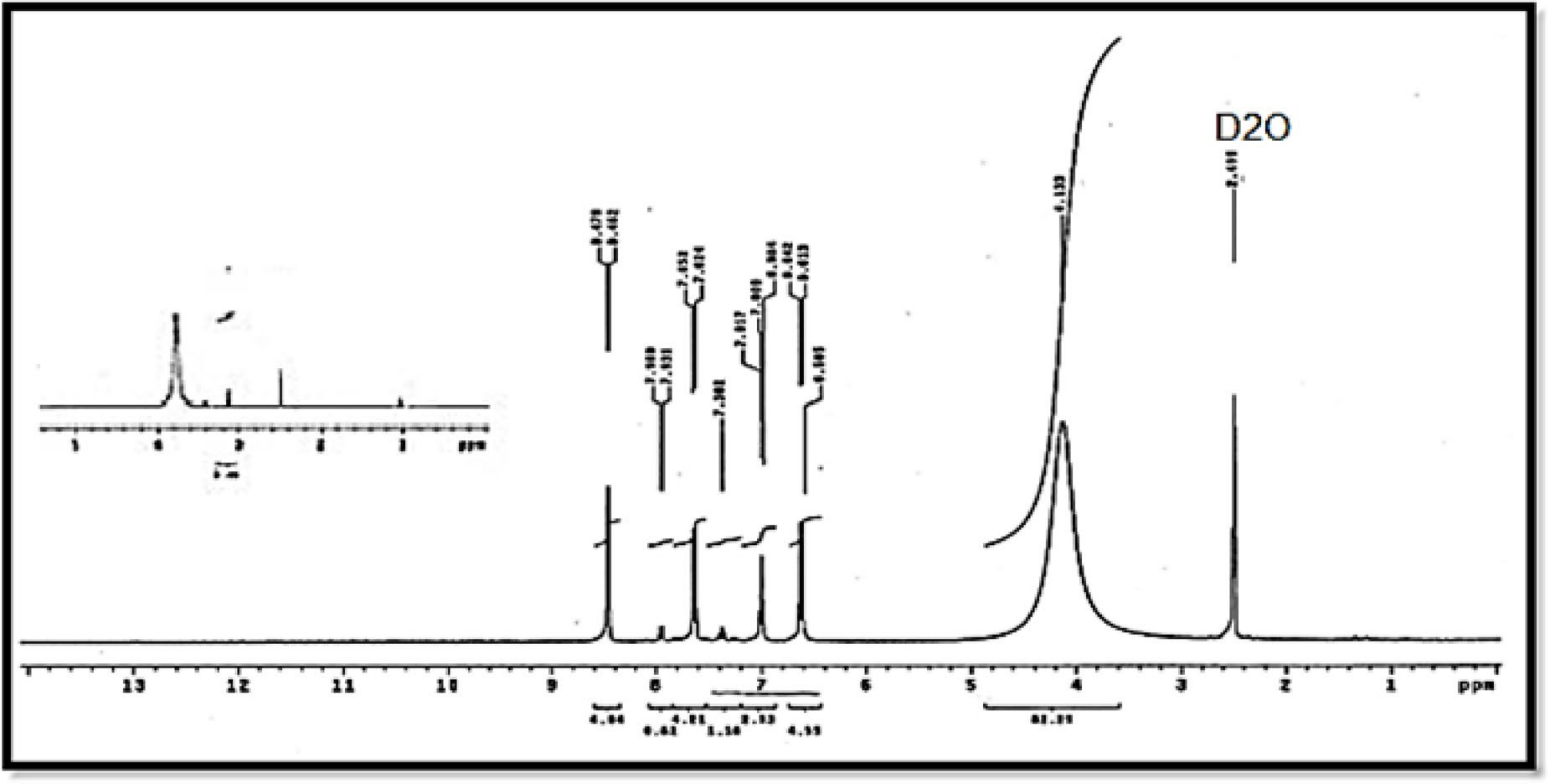
^1^H NMR diagram of Cd²⁺ metal complex.

According to the tabulated results in Table 5 and as shown in Figs. 4 and 5 the ¹H NMR spectra of ligand (L) in DMSO-d₆ show a broad signal at δ 4.85 ppm for the -SO₂NH proton of the enol/keto tautomer, exchangeable with D₂O, and a doublet at δ 8.48 ppm for the heterocyclic -CH proton. Aromatic protons of the pyrimidine, benzene, and nitrobenzene rings appear as multiple doublet–doublet signals between 6.6 and 7.6 ppm, consistent with the proposed ligand structure. Upon complexation with Cd²⁺ to form [(CdCl₂)₂(L)(H₂O)₄], the -SO₂NH proton shifts downfield to 5.959–11.21 ppm, indicating coordination and/or hydrogen-bonding interactions, while the heterocyclic -CH and aromatic protons show minimal shifts, suggesting they are not directly involved in binding. A new signal at δ 3.363 ppm confirms the presence of coordinated water molecules. These results indicate that Cd²⁺ binds primarily through the sulfonamide -NH/-OH group, leaving the aromatic and heterocyclic systems largely unaffected^20,21^.

**Table 5:** ¹H NMR data of ligand (L), and Its Cd(II) complex.

Compound	Pyrimidine protons	Benzene protons	Nitrobenzene protons	Heterocyclic -CH	Exocyclic -NH / -OH	Coordinated H_2_O
ligand (L)	6.602–7.02	6.98–7.50	7.474–7.649	8.48 (d, 3 H)	4.85 (s, 2 H)	--
Cd²⁺ complex	6.554–6.584	6.978–7.01	7.603–7.641	8.479 (d, 3 H)	5.959–11.21 (s, 2 H)	3.363

Chemical shifts δ (ppm), multiplicity (s = singlet, d = doublet), integration (H).

### ¹³C NMR spectrum of the free ligand

**Fig. 6:**
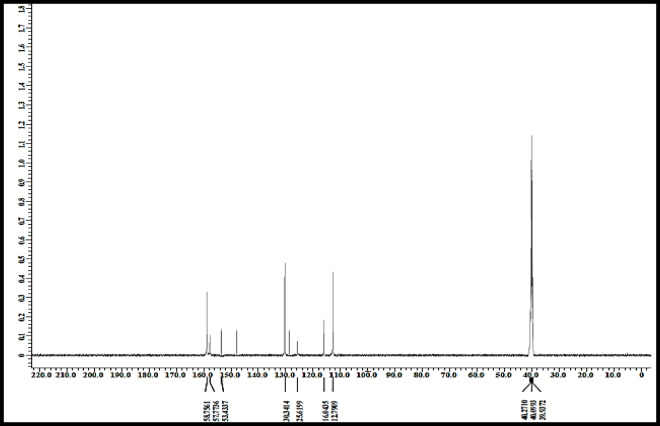
^13^C NMR data of free ligand.

The ¹³C NMR spectrum of the free ligand was recorded in DMSO-d₆ and exhibited well-resolved signals consistent with the proposed structure. A characteristic solvent peak was observed at approximately 40 ppm. The signal at 112.79 ppm was assigned to the pyrimidine ring carbon (C1). The resonance at 116.04 ppm was attributed to the ortho-benzene carbon (C2), while signals at 125.62, 129.00, and 130.34 ppm correspond to para- and meta-substituted benzene carbons (C3–C5). Furthermore, the downfield signals at 149.01 and 153.43 ppm were assigned to benzene carbons (C6 and C7) directly bonded to the nitrogen atoms of the phospha(V)azo moiety, indicating deshielding due to electron-withdrawing effects. The most downfield signals, observed at 157.77 and 158.76 ppm, were attributed to the C = N carbons (C8 and C9) of the pyrimidine ring. These assignments are in good agreement with previously reported data and support the successful formation of the proposed ligand structure, as represented in Fig. 6; Table 6^16,20–38^.

**Table 6:** ¹³C NMR data of the free ligand in DMSO-d₆.

Carbon atom	Chemical Shift δ (ppm)	Assignment
C1 (pyrimidine ring)	112.79	Pyrimidine carbon
C2 (ortho-benzene)	116.04	Aromatic carbon
C3 (para-benzene, attached to SO₂)	125.62	Aromatic carbon
C4 (meta-benzene)	129.00	Aromatic carbon
C5 (para-benzene)	130.34	Aromatic carbon
C6, C7 (benzene carbons attached to N of phospha(V)azo)	149.01, 153.43	Benzene carbons linked to nitrogen
C8, C9 (pyrimidine C = N)	157.77, 158.76	Pyrimidine C = N carbons
Solvent (DMSO-d₆)	40.0	Reference signal

### Thermogravimetric analysis (TGA)

**Fig. 7:**
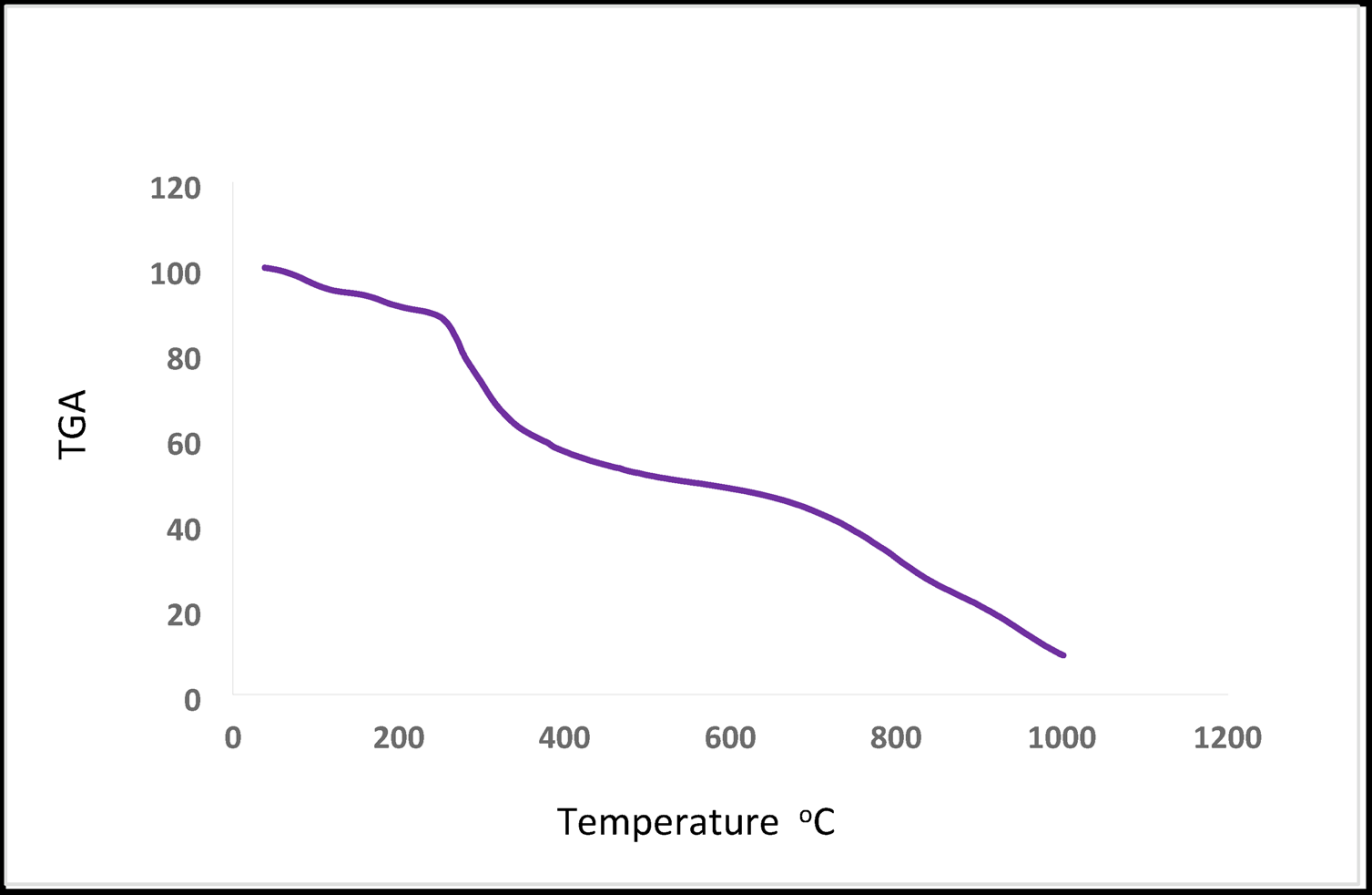
TGA Curve of Cu²⁺ complex.

**Fig. 8:**
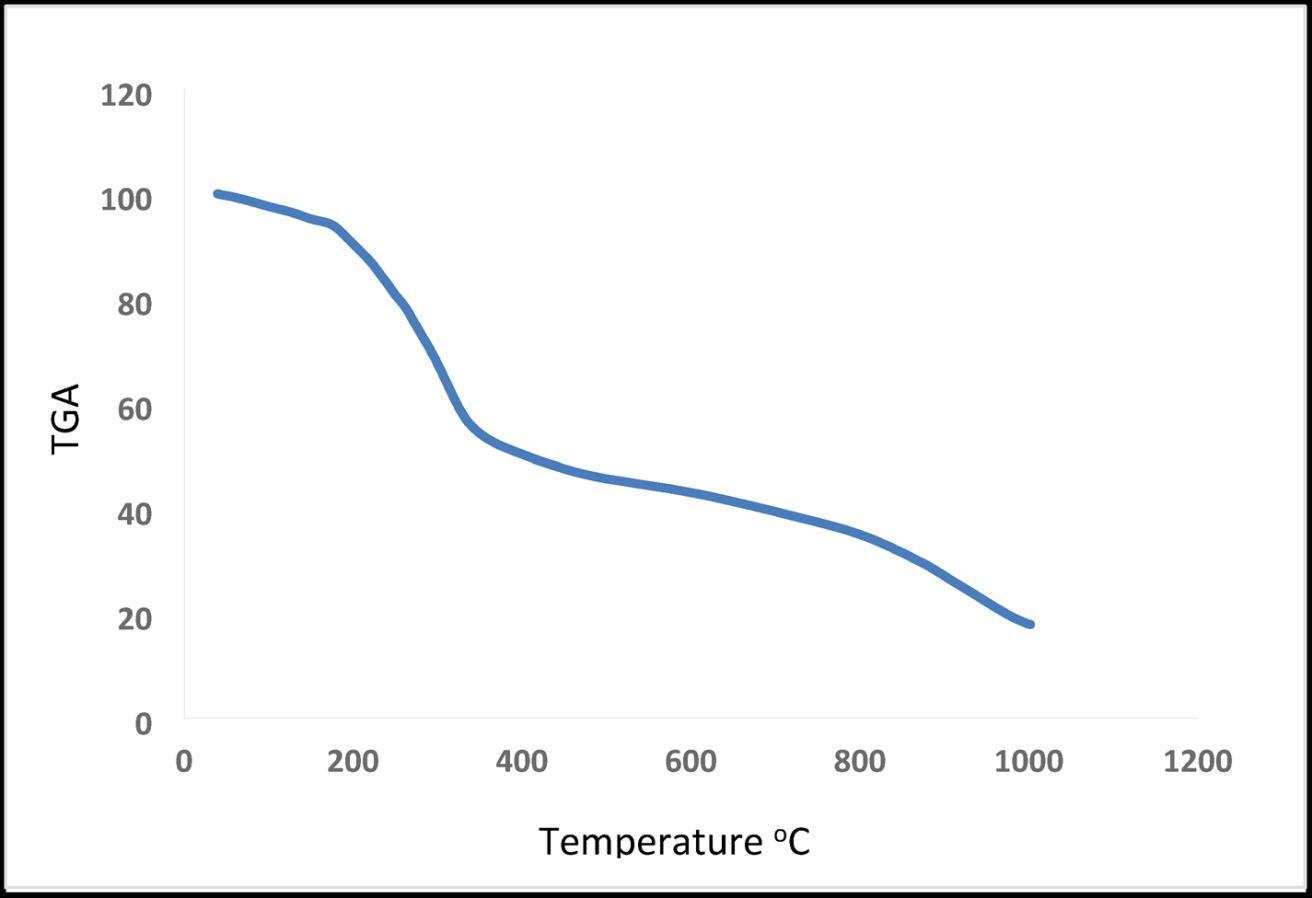
TGA Curve of Cd²⁺ complex.

Thermogravimetric analysis (TGA) is a valuable technique used to distinguish between lattice (crystalline) and coordinated water molecules in metal complexes, as well as to investigate their thermal decomposition behavior. As shown in Table 7; Figs. 7 and 8, the TGA curve of the complex Cu²⁺ complex shows three distinct decomposition steps over the temperature range 38–823 °C. The first stage (38–147 °C) corresponds to the loss of four coordinated water molecules, with an observed mass loss of 5.21% (calcd. 5.56%), confirming their coordination within the inner sphere. The second stage (147–459 °C) is attributed to the decomposition of the organic moiety C₂₀H₁₃Cl₄N₄O₄P₂S₂, with a mass loss of 47.70% (calcd. 48.22%). The third stage (462–823 °C) corresponds to the removal of the remaining organic fragment C₁₂H₁₃Cl₂N₆O₄, with a mass loss of 29.06% (calcd. 29.05%). The final residue is consistent with the formation of sTable 2CuCl₂, with a total mass loss of 81.97% (calcd. 82.83%). Similarly, the TGA curve of the complex Cd²⁺ complex exhibits three decomposition steps within the temperature range 166–608 °C. The first stage (166–199 °C) corresponds to the loss of four coordinated water molecules, with a mass loss of 5.38% (calcd. 5.17%). The second stage (199–274 °C) is assigned to the decomposition of the fragment C₁₈H₁₁N₄O₄P₂, with a mass loss of 30.04% (calcd. 29.23%). The third stage (274–608 °C) involves the elimination of 6HCl and 6HCN, leaving behind C₆H₁₀S₂, with a mass loss of 27.54% (calcd. 28.37%). The final residue corresponds to stable 2CdCl₂, with a total mass loss of 63.10% (calcd. 62.77%). These results confirm the proposed structures of the synthesized ligand and its Cu²⁺ and Cd²⁺ complexes, supporting the presence of coordinated water molecules and the stepwise thermal decomposition pattern^16,20,21,39^.

**Table 7:** Thermogravimetric of Cu(II) and Cd(II) complexes.

Complex	Temp. range (°C)	n*	Mass loss (%) Found (Calc.)	Total mass loss (%) Found (Calc.)	Assignment	Residue
Cu²⁺ complex	38–147 147–459 462–823	3	5.21 (5.56) 47.70 (48.22) 29.06 (29.05)	81.97 (82.83)	Loss of 4 H₂O (coordinated) Loss of C₂₀H₁₃Cl₄N₄O₄P₂S₂ Loss of C₁₂H₁₃Cl₂N₆O₄	2CuCl₂
Cd²⁺ complex	166–199 199–274 274–608	3	5.38 (5.17) 30.04 (29.23) 27.54 (28.37)	63.10 (62.77)	Loss of 4 H₂O Loss of C₁₈H₁₁N₄O₄P₂ Loss of 6HCl + 6HCN leaving C₆H₁₀S₂	2CdCl₂

n* : Number of decomposition step.

### X-ray diffractometer analysis

**Fig. 9:**
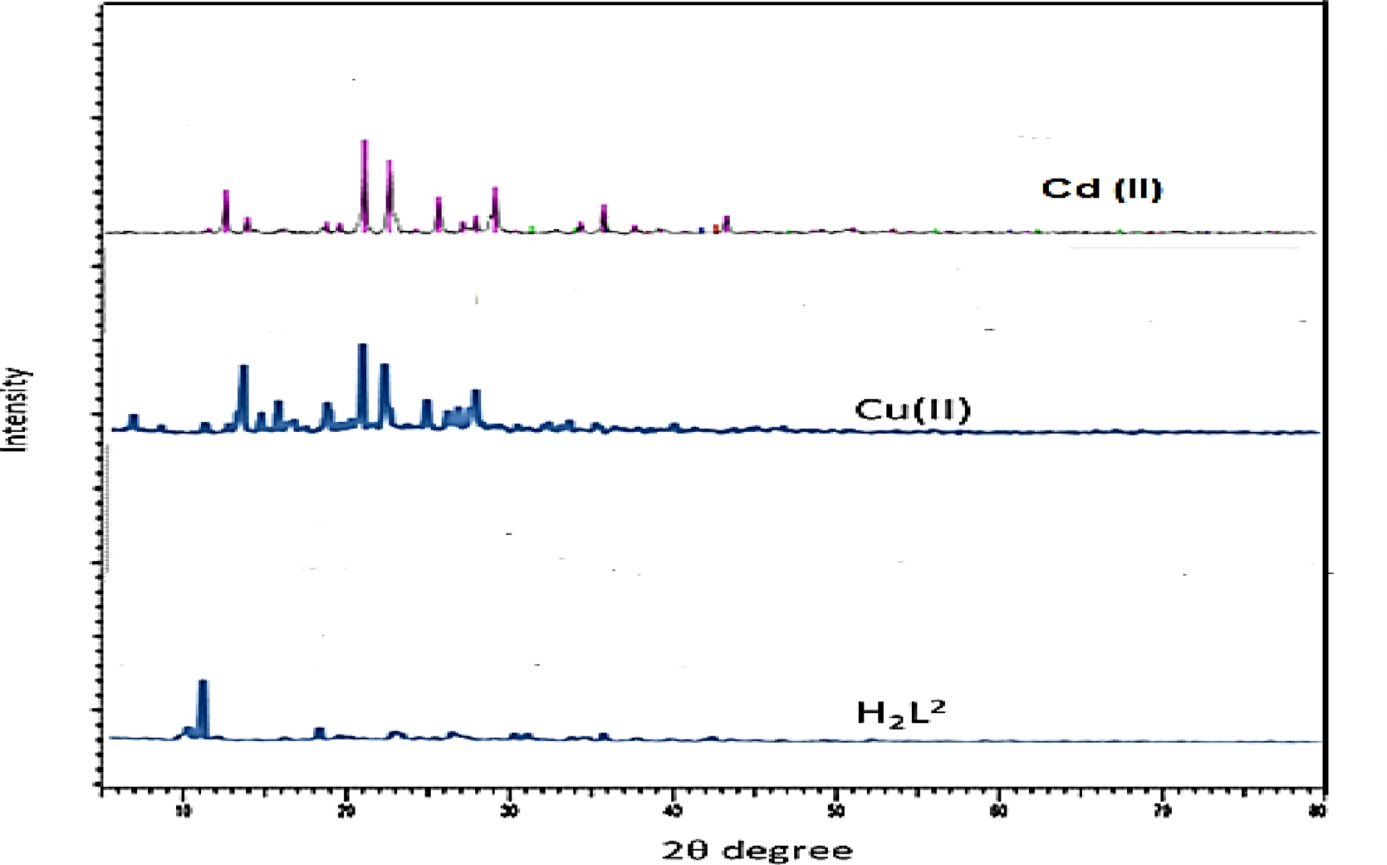
X-ray powder diffractogram of the free ligand, Cu²⁺ and Cd²⁺ metal complexes.

The crystalline structure of the synthetic ligand and its metal complexes, Cu²⁺ and Cd²⁺ complexes were investigated using a powder X-ray diffractometer. Figure 9 shows the powder XRD patterns of the free ligand and its metal complexes. The presence of sharp diffraction peaks indicates a high degree of crystallinity. Variations in the XRD patterns between the free ligand and the metal complexes are attributed to the incorporation of metal ions, which alters the crystal lattice. The average crystallite size of the samples was calculated using the Debye–Scherrer equation:

$$D = {{K\lambda } \mathord{\left/ {\vphantom {{K\lambda } {\left( {\beta \,\cos \theta } \right)}}} \right. \kern-\nulldelimiterspace} {\left( {\beta \,\cos \theta } \right)}}$$

where λ is the wavelength of the X-ray source (1.54 Å), θ is the Bragg diffraction angle corresponding to the (hkl) plane, and K is a constant equal to 0.94. The diffraction pattern of the free ligand shows 11 reflections in the range of 2θ = 21°–47°, with a maximum peak at 2θ = 21.17°, corresponding to a d-spacing of 4.19 Å. The Cu²⁺ complex exhibits 23 reflections in the range of 2θ = 12°–73°, with a maximum peak at 2θ = 21.18° (d = 4.19 Å). Meanwhile, the Cd²⁺complex displays 29 reflections between 2θ = 18°–59°, with a maximum peak at 2θ = 21.50° (d = 4.13 Å). The calculated average crystallite sizes are in the nanometer range, with values of 9.7–5.6 nm for the ligand and Cu²⁺complex, and 8.7 nm for the Cd²⁺complex. Slight shifts in peak positions, changes in intensity, and the appearance of new peaks confirm the formation of new coordination complexes. These observations are consistent with the data presented in Fig. 9. In conclusion, the sharp diffraction peaks confirm a high degree of crystallinity. Although the use of metal complexes in pharmaceutical synthesis has declined, combining classical coordination chemistry with modern analytical techniques such as X-ray diffraction and infrared spectroscopy offers promising opportunities for qualitative analytical applications.

### Application of the prepared cyclodiphosph(V)azane Ligand (L) and its Cu²⁺ and Cd²⁺ complexes as antimicrobial additives

#### Coating composition

The paint formulations were prepared according to our previous report^20,21^. through incorporating the prepared ligand, and its metal complexes with the ratio of 1.0 wt%, into topcoat paint formulation to compare it with the commercial sample without biocide, which supplied from the Pachine company, Table 7. lists the produced coating sample’s constituents. All the coated samples were allowed to completely dry for a few days before testing. Elcometer 415 thickness gauge was used to measure the thickness of dried coating films, and an average thickness of 60 ± 5 μm was obtained for the tested samples.

**Table 8:** Topcoat formulation and preparation steps.

Ingredients	F0(blank without biocide)	F1, Commercial sample	F2, based on prepared ligand(L)	F3, based on Cu²⁺complex	F4, based on Cd²⁺complex
Alkyd fast dray	20.0	20	20.0	20.0	20.0
Xylene	5.0	5.0	5.0	5.0	5.0
Stir for 3 min. for good mixing					
Anti-settling Gel	3.0	3.0	3.0	3.0	3.0
Stirring for 5 min. for completely miscible					
Fumed silica	0.5	0.5	0.5	0.5	0.5
Stirring for 5 min. for completely soluble					
Anti-terra U	1.0	1.0	1.0	1.0	1.0
Titanium dioxide	20.0	20.0	2.0	20.0	20.0
Talk powder	15.0	15.0	15.0	15.0	15.0
Xylene	7.0	7.0	7.0	7.0	7.0
CaCO_3_	6.0	6.0	6.0	6.0	6.0
High speed until reach to optimum fineness (becareful, Temperature should be up to 45 °C					
Low speed for the following					
Alkyd fast dray	15.0	15.0	15.0	15.0	15.0
Biocide	-----	1.0	1.0	1.0	1.0
Dryers	0.5	0.5	0.5	0.5	0.5
Xlene	5.0	5.0	5.0	5.0	5.0
Iso-butanol	2.5	2.5	2.5	2.5	2.5
Methyl Ethyl ketoxim	0.3	0.3	0.3	0.3	0.3
UV. Stabilizer	0.1	0.1	0.1	0.1	0.1
Total	100 ± 1.0	100 ± 1.0	100 ± 1.0	100 ± 1.0	100 ± 1.0

### Characterization of the prepared paint formulation

#### SEM analysis of the prepared topcoat formulations

**Fig. 10:**
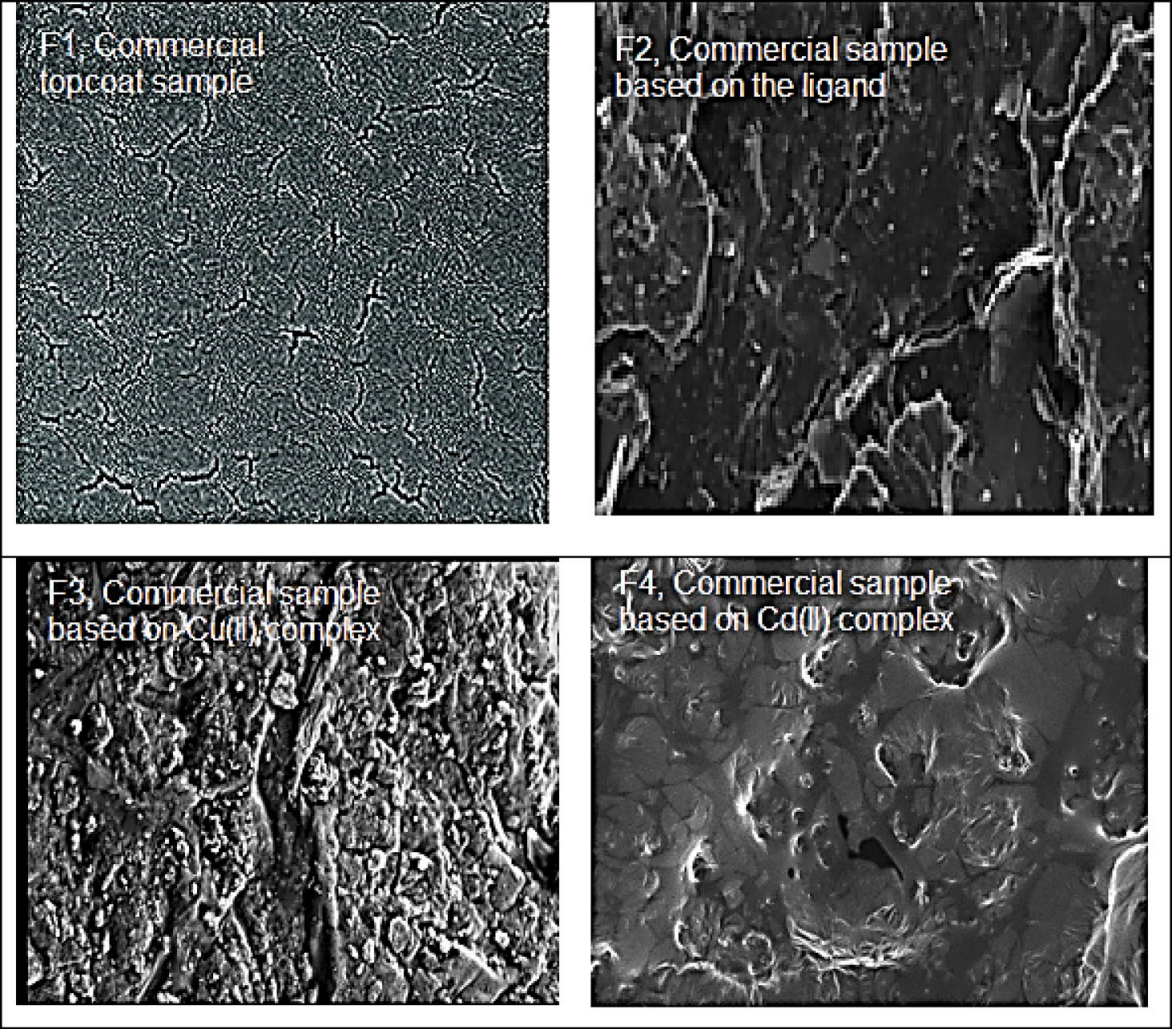
SEM image of (**a**) commercial topcoat formulation, (**b**) incorporated with ligand (L) (**c**) incorporated Cu²⁺metal complex, (**d**) incorporated Cd²⁺ metal complex.

As shown in Fig. 10, the cured paint film contains the integration of the prepared ligand and its metal complexes which exhibit no morphological abnormalities. This indicates that the mixed process was effective, the created paints were homogeneous and consistent, and the prepared coating were high-performance with superior rheology and fineness of grind. The shape of the net commercial top coating formula and its incorporation with the manufactured insecticide and antifungal additives were sufficiently observed using scanning electron microscopy (SEM). The SEM images of samples are shown in Fig. 10.

#### Physical-mechanical characteristics of the coatings

ASTM standards D1005, D523, D3363, D3359, D522, and D2794-93 were followed in measuring the dried films’ thickness, gloss, hardness, adhesion, flexibility, and impact resistance.

The influence of using the prepared compound by their incorporated with the topcoat formulation on the physical-mechanical characteristics were observed. So, based on the obtained results in Table 9, and the gloss, hardness, adhesion, and impact resistance results show that the mechanical characteristics of the coated films based on incorporated formulation are higher than the standard (commercial sample (F1) even the prepared sample without biocide (F0). For example, the gloss values were ranged from 80 to 96, hardness was increased from 7 to 9 H, adhesion improved from 4B – 5B, and impact resistance values also, increased from 1.3 to 2.5 (J). Even the viscosity was increased from 83 95 (KU). This may be due to the presence of complex structures with benzene rings, P, N, Cu, and Cd. And it was noticed that there’s no any effect on the flexibility and water immersion test, in which all samples were passed^20–22^.

**Table 9:** Physical and mechanical characteristics of the coated samples.

Formulation	Gloss at 60^0^	Scratch hardness (Pencil Hardness)	Adhesion	Flexibility	Impact (J)	Viscosity (KU)	Water immersion test (two days)
F0	80 ± 2.0	6 H	4B	Pass	1.3± 0.3	83 ± 1.2	Pass
F1	80 ± 1.0	7 H	4B	Pass	1.5± 0.3	851.2	Pass
F2	95 ± 2.0	9 H	5B	Pass	1.9± 0.2	901.3	Pass
F3	95 ± 2.0	9 H	5B	Pass	2.2± 0.1	951.3	Pass
F4	96 ± 1.0	9 H	5B	Pass	2.5± 0.2	951.3	Pass

F0, Topcoat formulation without biocide as blank for comparison.

F1: Commercial sample for comparison,

F2 topcoat formula incorporated with prepared ligand (L).

F3 topcoat formula incorporated prepared Cu²⁺ metal complex,

F4 topcoat formula incorporated the prepared Cd²⁺ metal complex.

6B < 5B < 4B < 3B < 2B < B < HB < F < H < 2 H < 3 H < 4 H < 5 H < 6 H < 7 H < 8 H 0B : Removed area is greater than 65%, 1B : 35–65% removed area, 2B : 15–35% removed area, 3B : 5–15% removed area, 4B : Less than 5%, 5B: 0% removed area.

#### Evaluation of the antimicrobial activity

**Fig. 11:**
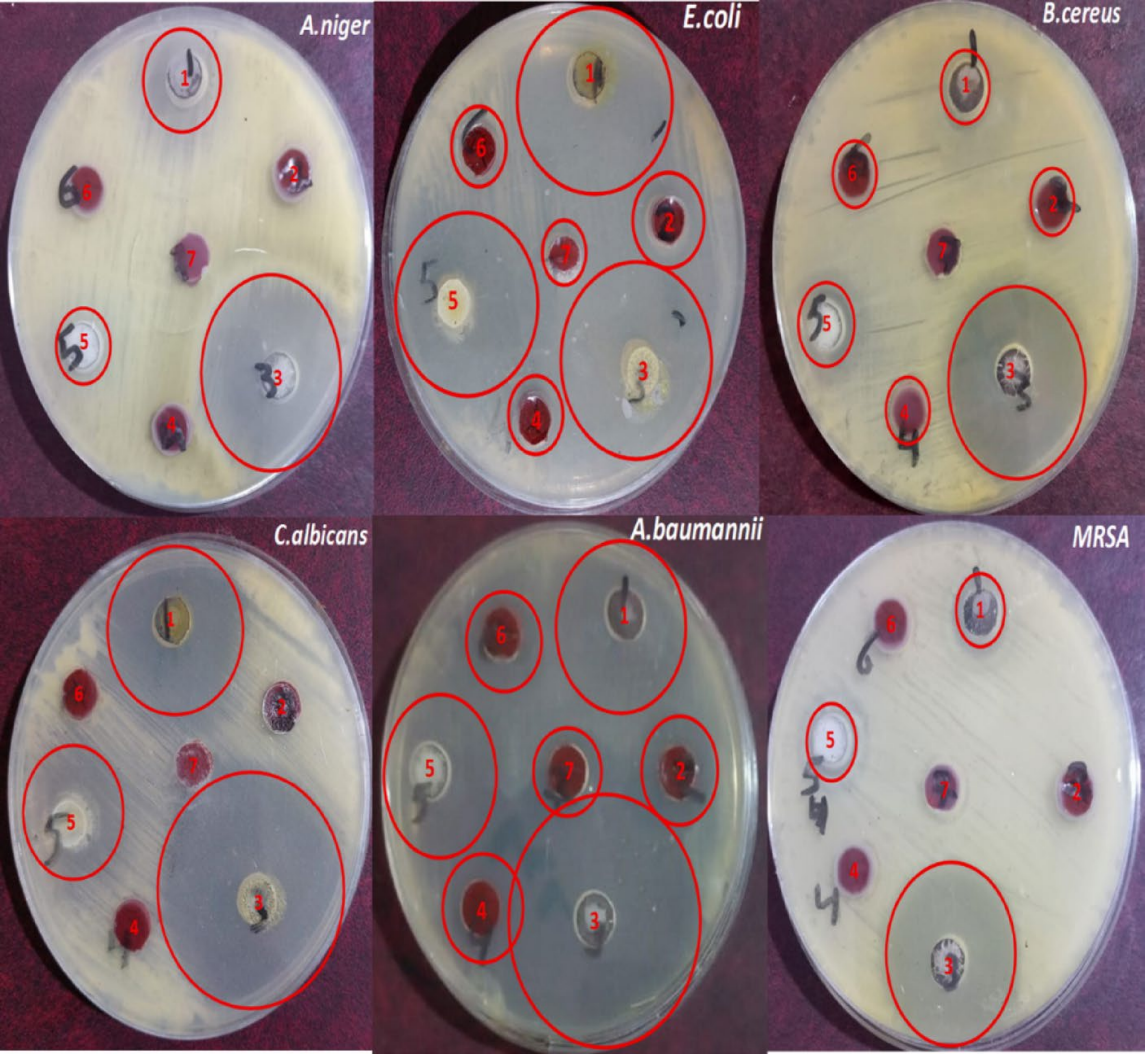
Antimicrobial activity of Cu²⁺ metal complex (1), Paint incorporated with Cu²⁺ metal complex, (2), Cd²⁺metal complex (3) paint incorporated with Cd²⁺ metal complex (4), ligand (L), (5), Paint incorporated with ligand (L). (6) Topcoat commercial sample) (7).

Marked differences in antimicrobial activity of copper, cadmium complexes, and their corresponding ligand—both alone and when incorporated into paint were observed. As show in Table 10; Fig. 11, Cadmium complex consistently produced the largest inhibition zones across most of the organisms, whereas Cupper and ligand produced moderate to low inhibition^22^. The antimicrobial assay revealed significant variability in inhibition zone diameters among the test samples. The cadmium complex exhibited the strongest antimicrobial effect, with inhibition zones reaching 46.0 ± 0.5 mm against *A. baumannii* and 42.0 ± 0.5 mm against *C. albicans*. Copper demonstrated moderate inhibitory activity, showing notable effects against *E. coli* (34.3 ± 0.8 mm) and *(A) baumannii* (31.3 ± 0.3 mm). The ligand alone showed relatively weak to moderate inhibition, with zones ranging from 12.8 to 34.8 mm. While the paint was incorporated with the prepared ligand and its Cu²⁺, Cd²⁺ metal complexes, we observed that there were reduced antimicrobial activity considerably. For incorporation with Cu²⁺metal complexes the obtained results showed inhibition, some activity remained—particularly against *(B) cereus*,* E. coli*, and *A. baumannii*. Minimal activity was detected in the blank paint (commercial sample), suggesting slight effects from paint additives. Regarding fungal organisms, Cadmium complex displayed dual antifungal activity against (C) albicans and *A.niger*, while Cupper and ligand produced moderate inhibition. Variability in inhibition zones among samples indicates differences in solubility, molecular size, and diffusion rates of the compounds. The presence of large inhibition zones does not necessarily indicate superior potency alone but may also reflect better diffusion characteristics in agar medium. Overall, the results demonstrate that Cadmium complex is a promising antimicrobial candidate with potent activity against both bacteria and fungi. The results indicate that the cadmium complex possesses broad-spectrum and highly potent antimicrobial activity, consistent with previous literature reporting that cadmium-based complexes generate oxidative stress and disrupt metal-dependent microbial enzymes. Copper also exhibited antimicrobial effects, which aligns with reports that copper ions can damage cellular membranes and interfere with metabolic pathways^40–42^, . The ligand alone displayed moderate antimicrobial effects, stronger against fungal isolates than bacteria, which may indicate selective biochemical interaction or partial synergy when complexed with metals. Finally, we can conclude that the antimicrobial activity of the coating formulations incorporated cyclodiphosph(V)azane ligand (L) and its Cu²⁺and Cd²⁺ is better than the commercial sample.

**Table 10:** The antimicrobial effect of the newly synthesized compounds and its incorporated with paint formulation.

Microorganisms	(Diameter of inhibition zone mm)							
	Pure Cu²⁺ metal complex	F4	Pure Cd²⁺ metal complex	F3	Pure ligand (L)	F2	F1	F0
MRSA	12.1 ± 0.1	0	31.8 ± 0.1	0	12.8 ± 0.1	0	0	0
*B. cereus*	13 ± 0.5	10.8 ± 0.4	34.5 ± 0.2	11.8±0.1	14 ± 0.2	12.7 ± 0.2	0	0
*E. coli*	34.3 ± 0.8	16.5 ± 0.2	36.6 ± 0.3	12.8±0.1	34.8 ± 0.4	13.6 ± 0.3	11.6 ± 0.3	0
*A. baumannii*	31.3 ± 0.3	17.5 ± 0.2	46 ± 0.5	19.5±0.2	26.3 ± 0.3	16.5 ± 0.2	15.3 ± 0.1	0
*C. albicans*	30 ± 0.5	0	42 ± 0.5	0	24.5 ± 0.2	0	0	0
*A. niger*	21 ± 0.5	0	34.3 ± 0.3	0	13.3 ± 0.3	0	0	0

F0, Topcoat formulation without biocide as blank for comparison.

F1: Commercial sample for comparison,

F2 topcoat formula incorporated with prepared ligand (L).

F3 topcoat formula incorporated prepared Cu²⁺ metal complex,

F4 topcoat formula incorporated the prepared Cd²⁺ metal complex.

### Determination of (MIC)

**Fig. 12:**
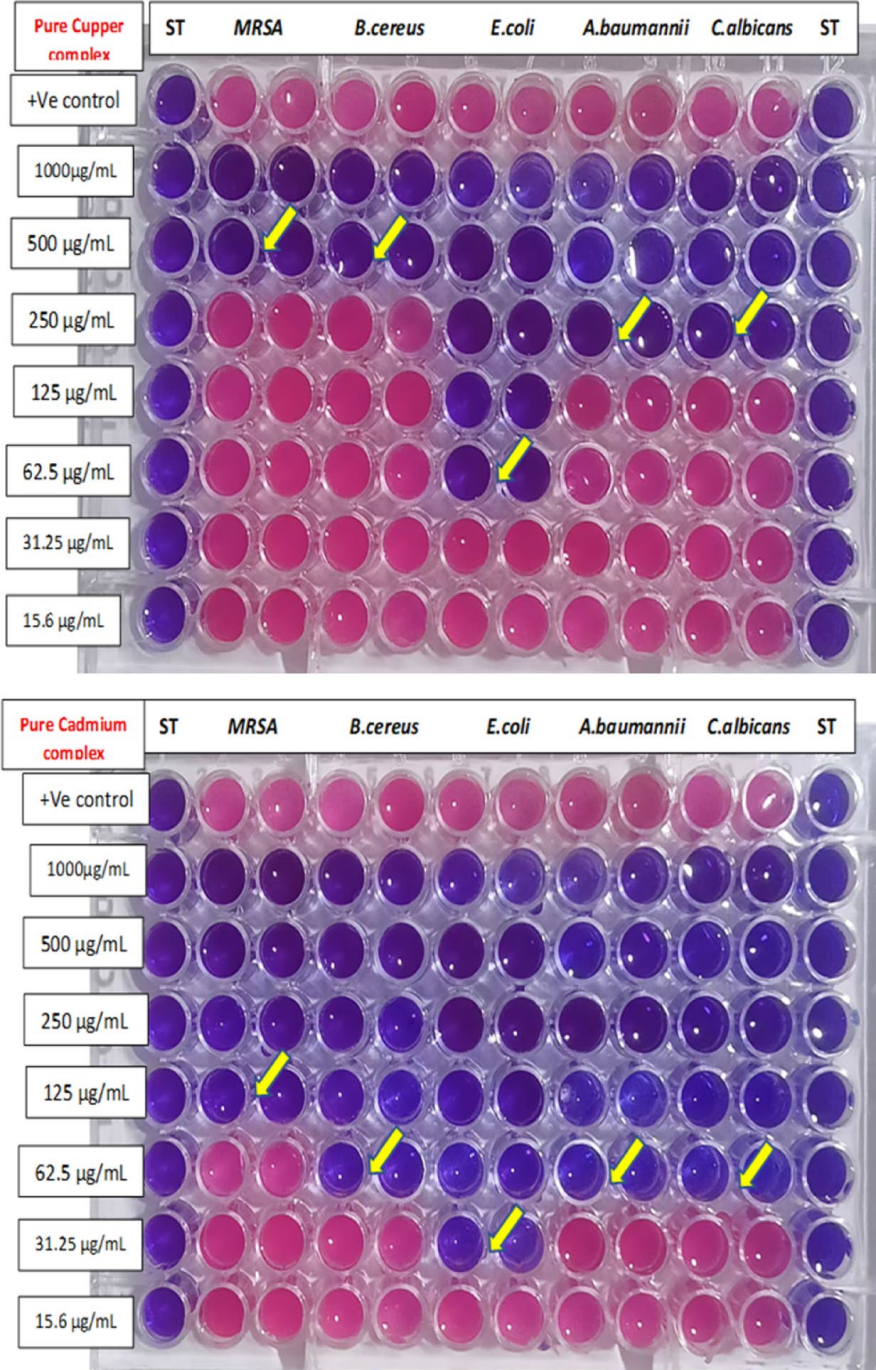

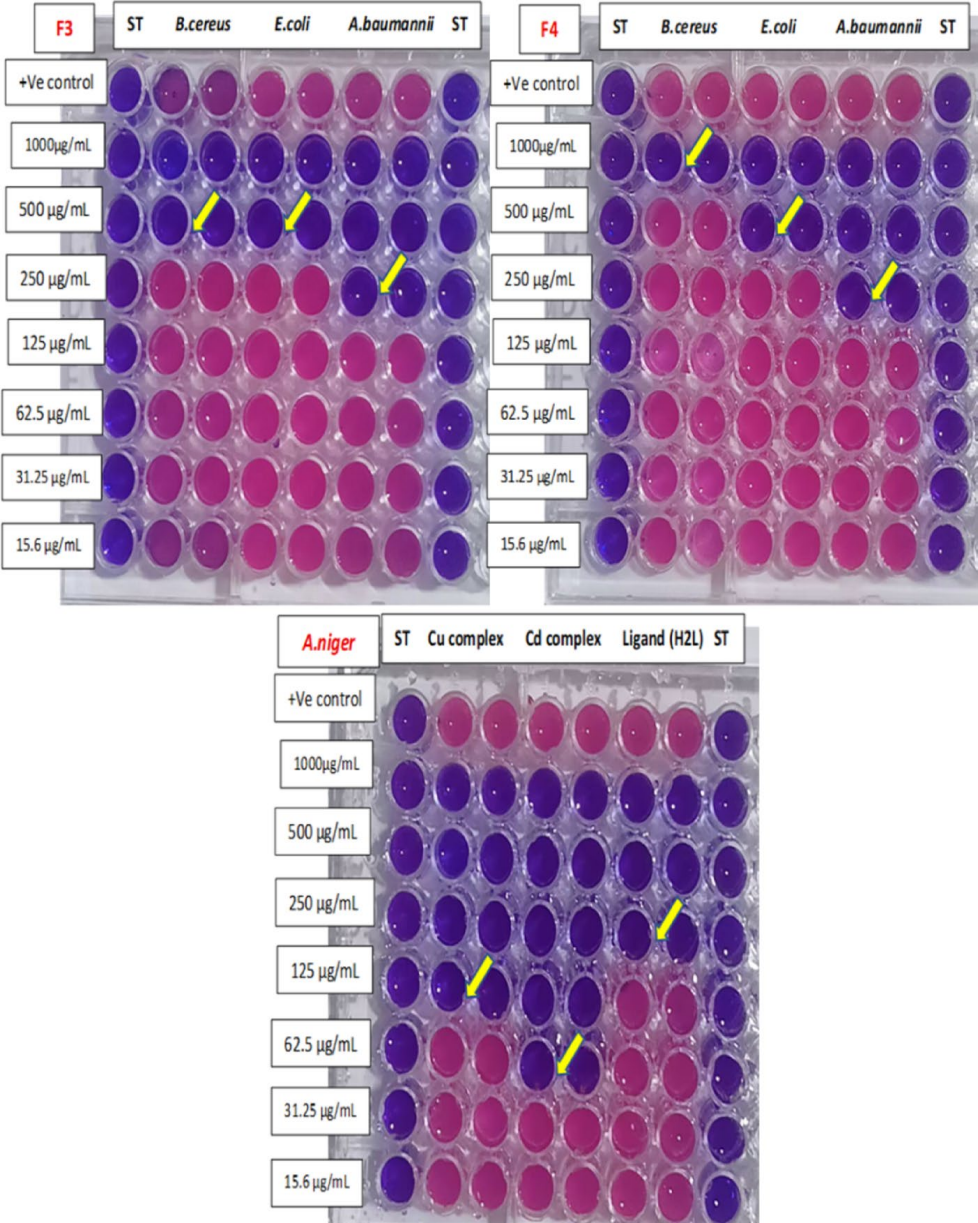
Determination of MIC of active compounds.

The MIC in Table (11) and Figure (12) reveals varying antimicrobial potencies among the pure Cu²⁺ metal complex, pure Cd²⁺metal complex, free ligand (L), and formulations F0-F4 against *MRSA*,* B. cereus*,* E. coli*,* A. baumannii*,* C. albicans*,* and (A) niger*. Pure copper complex shows moderate activity with MICs of 500 µg/mL against MRSA and *(B) cereus*, improving to 62.5 µg/mL for *E. coli and A. niger*, 250 µg/mL for *(A) baumannii* and *C. albicans.* F4 is less consistent, often at higher MICs like 1000 µg/mL for *(B) cereus* or inactive against MRSA and fungi. Pure Cadmium complex performs best among pure compounds at 31.25–125 µg/mL across most strains, while formulations generally less enhance potency, especially F4 and F3 at ≥ 250 µg/mL.

**Table 11:** MIC of the newly synthesized compounds and its incorporated with paint formulation against microbial pathogens.

Microorganisms	MIC(µg/mL)							
	Pure Cu²⁺ metal complex	F4	Pure Cd²⁺ metal complex	F3	Pure Ligand (L)	F2	F1	F0
MRSA	500	–	125	-	500	–	–	–
*B. cereus*	500	1000	62.5	500	250	250	–	–
*E. coli*	62.5	500	31.25	500	31.25	250	250	–
*A. baumannii*	250	250	62.5	250	250	125	125	–
*C. albicans*	250	–	62.5	–	250	–	–	–
*A. niger*	125	–	62.5	–	250	–	–	–

### Proposed mechanism of antimicrobial action of cyclodiphosph(V)azane derivatives within the coating matrix

The antimicrobial action of cyclodiphosph(V)azane derivatives in coatings can be summarized as a multifactorial mechanism:

1. Adhesion of the coating to microbial-contaminated surfaces.
2. Gradual release of metal complexes from the matrix.
3. Penetration into microbial cells (enhanced by lipophilicity).
4. Induction of oxidative stress and ROS generation.
5. Disruption of membranes and enzyme inhibition.
6. Cellular dysfunction and eventual microbial death.

The present study demonstrates that Cd²⁺ metal complex exhibits superior antimicrobial activity compared to the copper complex, free ligand (L), and their corresponding paint formulations. This difference in efficacy can be attributed to several physicochemical and biological factors related to metal ion properties, complex stability, and interaction with microbial cells. The enhanced activity of the cadmium complex may be primarily associated with its higher lipophilicity and membrane permeability. Metal complexation generally increases the lipophilic character of ligands, facilitating their diffusion through microbial cell membranes. Cd²⁺, with its relatively larger ionic radius and softer acid character, tends to form more stable complexes with donor atoms such as nitrogen and sulfur. This stability can enhance the ability of the complex to interact with vital intracellular targets, including proteins and nucleic acids. Furthermore, cadmium ions are known to disrupt enzymatic systems by binding to thiol (-SH) groups in proteins, leading to enzyme inactivation and cellular dysfunction. This may explain the consistently low MIC values observed across both Gram-positive, Gram-negative bacteria, and fungal strains. In contrast, the copper complex showed comparatively moderate activity, which may be due to its redox-active nature. Although copper can generate reactive oxygen species (ROS) that contribute to antimicrobial effects, its complexes are often less stable under biological conditions. This can lead to partial dissociation before reaching intracellular targets, thereby reducing overall efficacy. Additionally, copper ions may be more readily regulated or expelled by microbial defense mechanisms, such as efflux pumps and metal homeostasis systems, further limiting their antimicrobial potency. The free ligand (L) exhibited lower activity than its metal complexes, supporting the concept of chelation theory, where coordination with metal ions enhances biological activity. Upon complexation, the polarity of the metal ion is reduced through partial sharing of its positive charge with ligand donor groups, increasing lipophilicity and facilitating penetration into microbial cells. Without metal coordination, the ligand lacks this enhanced transport capability and target interaction efficiency. A notable observation in this study is the reduction in antimicrobial activity upon incorporation into paint formulations (F0–F4). This decrease can be attributed to several factors. First, the physical entrapment of active compounds within the paint matrix likely limits their diffusion and availability at the microbial interface. The polymeric components of the paint may act as a barrier, slowing the release rate of the active agents and thereby reducing their immediate antimicrobial effect. Second, possible interactions between the metal complexes and paint constituents (e.g., binders, pigments, or additives) may alter the chemical stability or reactivity of the active compounds. Such interactions could lead to partial deactivation or reduced accessibility of functional groups necessary for antimicrobial action. Additionally, the heterogeneous distribution of active compounds within the formulation may contribute to inconsistent activity, as observed particularly with F3 and F4. Lower surface exposure of the active species reduces effective contact with microbial cells, resulting in higher MIC values or complete inactivity in some cases. Finally, the controlled-release nature of coatings, while beneficial for long-term applications, may not be adequately captured in standard MIC assays, which are designed to measure immediate antimicrobial effects in solution. Thus, the apparent activity in formulated systems does not necessarily negate their practical utility but rather reflects differences in testing conditions. In summary, the superior antimicrobial performance of the cadmium complex is likely due to its favorable stability, lipophilicity, and strong interaction with microbial biomolecules, while the reduced activity in paint formulations arises from limited diffusion, matrix interactions, and reduced bioavailability. These findings highlight the importance of considering both chemical composition and delivery system when designing effective antimicrobial materials [43-48].

### Quantitative analysis and statistical validation of antimicrobial activity

Statical analysis was conducted using Minitab Statical Software, version 18 (Minitab LLC, State College, PA, USA; https://www.minitab.com), extended with a descriptive statistics package. To ensure the reliability and reproducibility of the antimicrobial results, all experiments were conducted in triplicate (*n* = 3), and the inhibition zone diameters were expressed as mean ± standard deviation (SD), as presented in Table 10. The relatively small SD values (± 0.1–0.8 mm) indicate high precision and good repeatability of the agar well diffusion measurements.

## Conclusions

A novel cyclodiphosph(V)azane sulfonamide ligand and its Cu²⁺ and Cd²⁺ metal complexes were successfully synthesized and characterized. When incorporated into paint formulations, these compounds significantly enhanced coating performance, including gloss (80–95), hardness (7–9 H), adhesion (4B–5B), and impact resistance (1.5–2.2 J). Antibacterial testing against six microbial strains showed that the Cd²⁺ metal complex exhibited the strongest activity (inhibition zones 31.8–46.0 mm), while the Cu(II) complex had moderate effects and the ligand showed selective inhibition. Although paint incorporation slightly reduced antimicrobial efficacy, significant activity remained against *E. coli*, *B. cereus*, and *A. baumannii*. These results demonstrate that locally prepared phosphazane ligands and their metal complexes can replace expensive imported antibacterial additives, improving coating durability and offering cost-effective, high-performance antimicrobial paints.

There is no conflict of interest in this paper.

## Data Availability

The datasets used and analyzed during the current study are available from the corresponding author upon reasonable request.
